# Short-term pyrrolidine dithiocarbamate administration attenuates cachexia-induced alterations to muscle and liver in *Apc^Min/+^* mice

**DOI:** 10.18632/oncotarget.10699

**Published:** 2016-07-19

**Authors:** Aditi A. Narsale, Melissa J. Puppa, Justin P. Hardee, Brandon N. VanderVeen, Reilly T. Enos, E. Angela Murphy, James A. Carson

**Affiliations:** ^1^ Department of Exercise Science, University of South Carolina, Columbia, South Carolina, USA; ^2^ Department of Pathology, Microbiology & Immunology, School of Medicine, University of South Carolina, Columbia, South Carolina, USA; ^3^ Center for Colon Cancer Research, University of South Carolina, Columbia, South Carolina, USA

**Keywords:** hepatomegaly, muscle atrophy, protein turnover, inflammation, metabolism

## Abstract

Cancer cachexia is a complex wasting condition characterized by chronic inflammation, disrupted energy metabolism, and severe muscle wasting. While evidence in pre-clinical cancer cachexia models have determined that different systemic inflammatory inhibitors can attenuate several characteristics of cachexia, there is a limited understanding of their effects after cachexia has developed, and whether short-term administration is sufficient to reverse cachexia-induced signaling in distinctive target tissues. Pyrrolidine dithiocarbamate (PDTC) is a thiol compound having anti-inflammatory and antioxidant properties which can inhibit STAT3 and nuclear factor κB (NF-κB) signaling in mice. This study examined the effect of short-term PDTC administration to *Apc^Min/+^* mice on cachexia-induced disruption of skeletal muscle protein turnover and liver metabolic function. At 16 weeks of age *Apc^Min/+^* mice initiating cachexia (7% BW loss) were administered PDTC (10mg/kg bw/d) for 2 weeks. Control *Apc^Min/+^* mice continued to lose body weight during the treatment period, while mice receiving PDTC had no further body weight decrease. PDTC had no effect on either intestinal tumor burden or circulating IL-6. In muscle, PDTC rescued signaling disrupting protein turnover regulation. PDTC suppressed the cachexia induction of STAT3, increased mTORC1 signaling and protein synthesis, and suppressed the induction of Atrogin-1 protein expression. Related to cachectic liver metabolic function, PDTC treatment attenuated glycogen and lipid content depletion independent to the activation of STAT3 and mTORC1 signaling. Overall, these results demonstrate short-term PDTC treatment to cachectic mice attenuated cancer-induced disruptions to muscle and liver signaling, and these changes were independent to altered tumor burden and circulating IL-6.

## INTRODUCTION

Cachexia is a severe wasting syndrome seen with the later stages of chronic diseases such as rheumatoid arthritis, COPD, AIDS, and cancer [1]. It has been estimated that approximately half of cancer patients will suffer from cachexia, with 20% of cancer-related deaths attributed to cachexia [2]. Once developed, cachectic symptoms can persists independent of the disease, and while current treatments effectively improve lifespan, treatments do little to improve quality of life in patients [1, 3]. While treatments that impede cachexia are important to reduce morbidity and mortality in cancer patients, there are currently no FDA approved treatments for cancer cachexia. This is in part due to the complex nature of the disease and the severity of cachectic symptoms varying between patients [4]. Cancer cachexia is characterized by chronically elevated systemic inflammation, severe hypermetabolism, and the loss of skeletal muscle [5, 6]. Skeletal muscle atrophy contributes to enhanced treatment toxicity, decreased quality of life, and increased morbidity and mortality in cancer patients [7]. Moreover, tumor-induced metabolic disruption in organs such as the liver can also contribute to hypermetabolism and wasting processes [5]. Since chronically elevated cytokines can promote systemic organ dysfunction, targeting systemic inflammation may improve indices of cachexia progression in cancer patients [8]. However, whether inflammatory signaling inhibition can improve cachexia progression after significant weight loss has occurred has not been widely examined.

The small thiol compound, pyrrolidine dithiocarbamate (PDTC) has been shown to have both anti-inflammatory and antioxidant properties [9–11]. PDTC can inhibit the activation of interleukin-6 (IL-6) signaling target, signal transducer and activator of transcription 3 (STAT3), and its association with transcriptional co-activators forkhead box O (FOXO) and CCAAT/enhancer binding protein beta (C/EBPβ) [10]. In addition, PDTC has been shown to suppress inflammatory processes through the inhibition of nuclear factor κB (NF-κB) signaling [12–14]. Moreover, gene expression related to protein translation and oxidative capacity can be upregulated by PDTC treatment in hepatocarcinoma (HepG2) cells [15, 16]. Given that the previously mentioned signaling pathways are disrupted in several wasting conditions, there is growing interest to determine the therapeutic potential of PDTC treatment. Indeed, PDTC treatment has been shown to improve muscle function and regeneration in mdx mice [17]. Related to cancer cachexia, PDTC decreased tumor NF-κB DNA binding, which led to reduced circulating IL-6 and the attenuation of muscle mass loss in colon-26 (C26) tumor-bearing mice [18]. Our laboratory has previously demonstrated PDTC treatment decreased STAT3 and NF-κB phosphorylation in both the *Apc^Min/+^* and Lewis lung carcinoma (LLC) tumor-bearing mice [19, 20], and in C2C12 myotubes incubated with LLC conditioned medium [19, 21]. Interestingly, a single PDTC dose stimulated mTOR signaling and mitochondrial protein expression in cachectic skeletal muscle [20]. While these studies provide initial evidence to the potential therapeutic benefits to inhibiting systemic inflammation on skeletal muscle, whether PDTC treatment disrupts protein and metabolic signaling pathways in other tissues during cancer cachexia progression has yet to be examined.

Systemic inflammation associated with cancer can contribute to cachexia progression through the disruption of multiple organ homeostasis [8]. While several cytokines have been implicated to mediate muscle wasting during cancer cachexia, IL-6 has been shown to play a critical role in cachexia progression in both human patients and in several preclinical cancer models [8, 22, 23]. The *Apc^Min/+^* mouse exhibits an IL-6-dependent cachexia, which has a slow onset and progression over a longer time period when compared to other preclinical cachexia models [24, 25]. This makes the *Apc^Min/+^* mouse advantageous for studies initiating treatment after the development of cachexia, which has clinical relevance to the human patient [1, 26]. Skeletal muscle and liver are two metabolically active tissues known to be impacted during cancer cachexia [8, 27]. We have found systemic IL-6 adversely affects skeletal muscle protein turnover and liver metabolism during the progression of cachexia [27, 28]. Activation of STAT3 signaling, a downstream mediator of the IL-6 family of cytokines signaling, can disrupt muscle protein turnover during cachexia [20, 27, 29]. In contrast to skeletal muscle, increased liver STAT3 activity was independent of cachexia severity in the *Apc^Min/+^* mouse [28]. While evidence in pre-clinical cancer cachexia models have determined that different systemic inflammatory inhibitors, including IL-6, can attenuate several characteristics of cachexia [20, 27, 30], there is a limited understanding of the effect of these inhibitors after cachexia has developed, and whether short-term administration is sufficient to reverse cachexia-induced signaling in distinct target tissues. Therefore, the purpose of this study was to determine the effect of short-term PDTC administration to cachectic *Apc^Min/+^* mice on the cachexia-induced disruption of skeletal muscle protein turnover and liver metabolic function. We hypothesized that PDTC administration would improve the cachexia disruption of muscle protein turnover and liver metabolic function in *Apc^Min/+^* mice. To test this hypothesis, *Apc^Min/+^* mice that had initiated cachexia were administered PDTC daily for 2 weeks and indices of cachexia progression related to systemic inflammation, muscle protein turnover, and liver metabolic function were examined. The results demonstrate PDTC treatment improved muscle protein turnover and liver glycogen content independent to changes in circulating IL-6 and tumor burden.

## RESULTS

### Effect of PDTC treatment on cachexia progression in *Apc^Min/+^* mice

**Figure 1:**
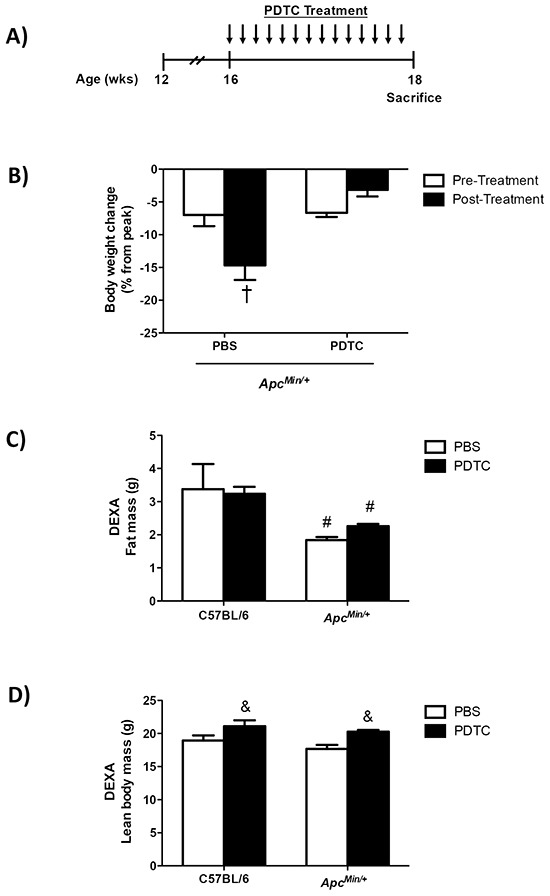

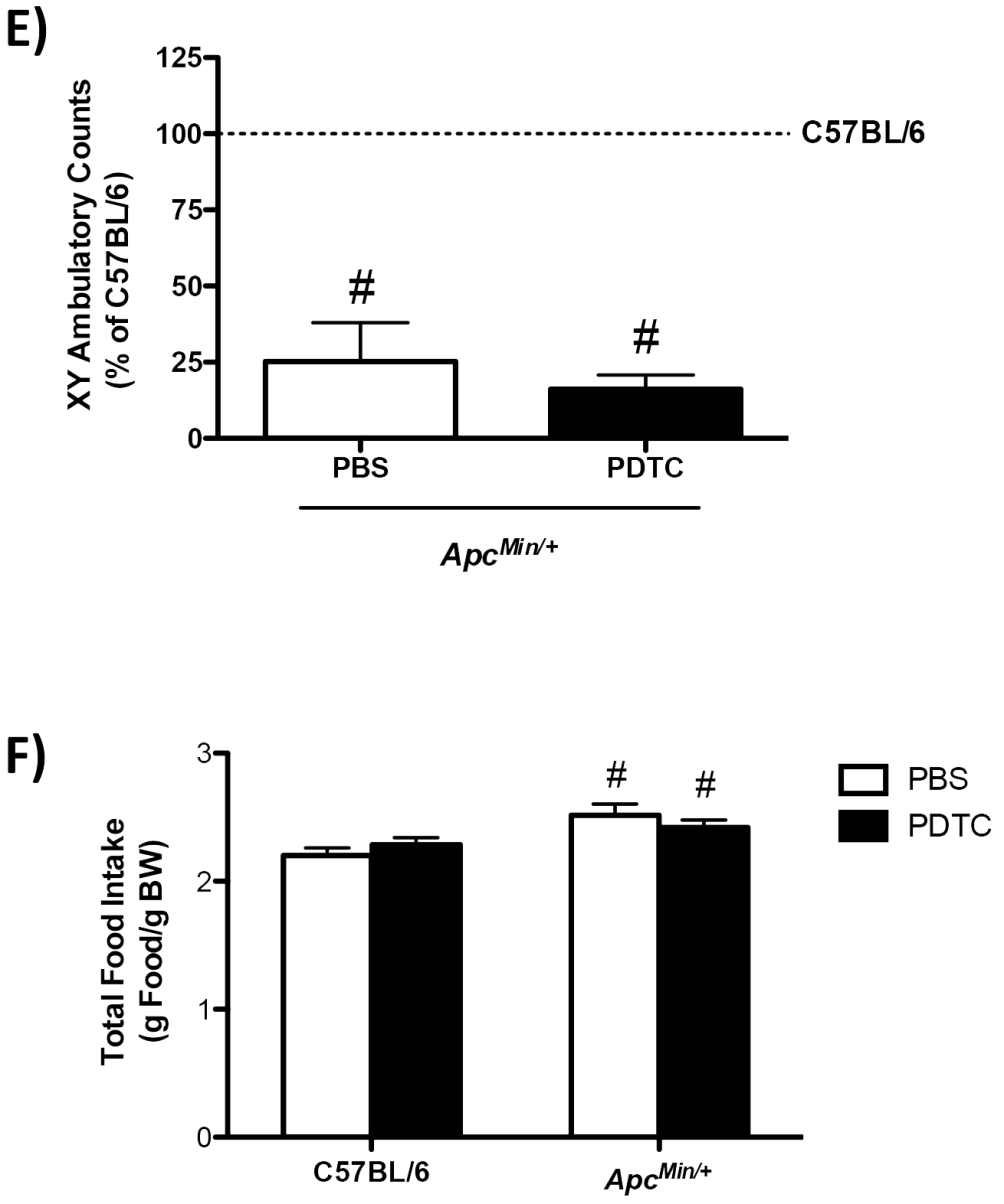
The effect of PDTC treatment on cachexia progression in *Apc^Min/+^* mice **A.** Experimental Design. At 16 weeks of age C57BL/6 and *Apc^Min/+^* mice were randomized to receive PBS or PDTC (10 mg/kg body weight/day) via intraperitoneal injection for 2 weeks. Mice were sacrificed at 18 weeks of age following a 5 hr fast. **B.** The percentage body weight loss pre- and post-treatment in *Apc^Min/+^* mice. **C.** Total fat mass measured by DEXA in C57BL/6 and *Apc^Min/+^* mice. **D.** Total lean body mass measured by DEXA in C57BL/6 and *Apc^Min/+^* mice. **E.** XY ambulatory counts in C57BL/6 and *Apc^Min/+^* mice. Data are expressed as the percentage of C57BL/6 + PBS. **F.** Total food intake during the 2-wk treatment period in a second cohort of *Apc^Min/+^* mice that had initiated body weight loss (C57BL/6 + PBS: N=5, C57BL/6 + PDTC: N=6, *Apc^Min/+^* + PBS: N=5, *Apc^Min/+^* + PDTC: N=7). Values are means ± standard error. Significance was set at P<0.05. † = signifies difference to all groups. & = signifies main effect of PDTC treatment. # = signifies main effect of *Apc^Min/+^* genotype. * = signifies difference from PBS treatment within genotype. Abbreviations: wks, weeks. mg, milligram. PBS, phosphate buffered saline. PDTC, pyrrolidine dithiocarbamate.DEXA, Dual-energy X-ray absorptiometry.

The effects of PDTC treatment on cachexia progression were examined in *Apc^Min/+^* mice. There were no differences in peak body weight between C57BL/6 and *Apc^Min/+^* mice prior to PDTC treatment (Table 1). *Apc^Min/+^* mice had initiated cachexia prior to treatment (Table 1; Figure 1B). Control *Apc^Min/+^* mice continued to lose body weight during the treatment period, while mice receiving PDTC had no further body weight decrease (Table 1; Figure 1B). There was no effect of PDTC treatment on body weight in C57BL/6 mice. Total hindlimb muscle mass was reduced in *Apc^Min/+^* mice, but PDTC treatment increased hindlimb muscle mass irrespective of genotype (Table 1). While gastrocnemius muscle mass was reduced in *Apc^Min/+^* mice, there was a strong trend for PDTC treatment to increase gastrocnemius muscle mass regardless of genotype (P=0.06; Table 1). Total fat mass measured by DEXA and epididymal fat mass was reduced in *Apc^Min/+^* mice, and there was no effect of PDTC treatment (Table 1; Figure 1C). Interestingly, PDTC treatment increased lean body mass irrespective of genotype (Figure 1D). Liver weight was increased in *Apc^Min/+^* mice, and PDTC treatment increased liver weight irrespective of genotype (Table 1). Remarkably, PDTC treatment increased heart mass irrespective of genotype (Table 1). There was no effect of PDTC treatment on reduced testes weight in *Apc^Min/+^* mice (Table 1). While tibia length was similar between C57BL/6 and *Apc^Min/+^* mice, PDTC treatment increased tibia length irrespective of genotype (Table 1). Cage activity was monitored two days prior to sacrifice to determine if improvements in body weight and muscle mass were associated with alterations in physical activity levels. While cage activity was reduced in *Apc^Min/+^* mice, there was no effect of PDTC treatment (Figure 1E). Interestingly, PDTC treatment increased cage activity in C57BL/6 mice (PBS: 15321 ± 5036 counts vs PDTC: 28927 ± 5376 counts; Average ± SE). A second cohort of mice initiating cachexia was treated for two weeks to determine the effects of PDTC treatment on food intake. Similar to the previous cohort, these mice had initiated cachexia prior to treatment. Control *Apc^Min/+^* mice continued to lose body weight during the treatment period, while mice receiving PDTC had no further body weight decrease (Supplementary Figure S1A). There was a main effect of PDTC treatment to increase total food intake during the treatment period irrespective of genotype (Supplementary Figure S1B). However, this likely reflects changes in body weight during the treatment period, as there were no differences in food intake when corrected for body weight. In fact, *Apc^Min/+^* mice demonstrated increased relative food intake over the treatment period compared to C57BL/6 (Figure 1F). Collectively, these data demonstrate that PDTC treatment attenuated body weight and muscle mass loss independent to improvements in physical activity level and food intake, however there was growth to organs such as the liver and heart in *Apc^Min/+^* mice.

**Table 1:** The effect of PDTC treatment on cachexia progression in *Apc^Min/+^* mice

	C57BL/6		*Apc^Min/+^*	
	PBS	PDTC	PBS	PDTC
**Body weight, g**				
***Peak***	25.3 ± 1.1	28.2 ± 1.3	24.3 ± 0.5	25.4 ± 0.4
***Pre-Treatment, 16 wks***	25.3 ± 1.1	28.2 ± 1.3	22.6 ± 0.5^*^^#^	23.8 ± 0.5^*^^#^
***Post-Treatment, 18 wks***	25.7 ± 1.2	27.5 ± 1.3	21.0 ± 0.4^*^^†^^#^	24.6 ± 0.3^#^
**Total hindlimb mass, mg**	305 ± 16	342 ± 156^&^	196 ± 20^#^	230 ± 7^#^^&^
**Gastrocnemius, mg**	135 ± 4	142 ± 6	86 ± 7^#^	97 ± 3^#^
**Epididymal fat, mg**	387 ± 117	429 ± 48	34 ± 34^#^	135 ± 43^#^
**Liver, g**	1.0 ± 0.06	1.3 ± 0.09^&^	1.4 ± 0.06^#^	1.9 ± 0.07^#^^&^
**Heart, mg**	105 ± 5	120 ± 54^&^	109 ± 4	136 ± 7^&^
**Testes, mg**	197 ± 6	206 ± 8	146 ± 19^#^	150 ± 12^#^
**Tibia length, mm**	16.6 ± 0.2	17.2 ± 0.1^&^	16.6 ± 0.2	16.9 ± 0.1^&^
**No. of mice**	5	5	6	6

Values are means ± standard error. Peak body weight was determined as the highest body weight achieved prior to treatment. Abbreviations: PBS, phosphate buffered saline. PDTC, pyrrolidine dithiocarbamate. g, grams. mg, milligram. mm, millimeter. No., number of animals.

* signifies difference from peak body weight measurement.

† signifies difference from pre body weight measurement.

# signifies main effect of *Apc^Min/+^* genotype.

& signifies main effect of PDTC treatment.

### Effect of PDTC treatment on systemic inflammation in *Apc^Min/+^* mice

**Figure 2:**
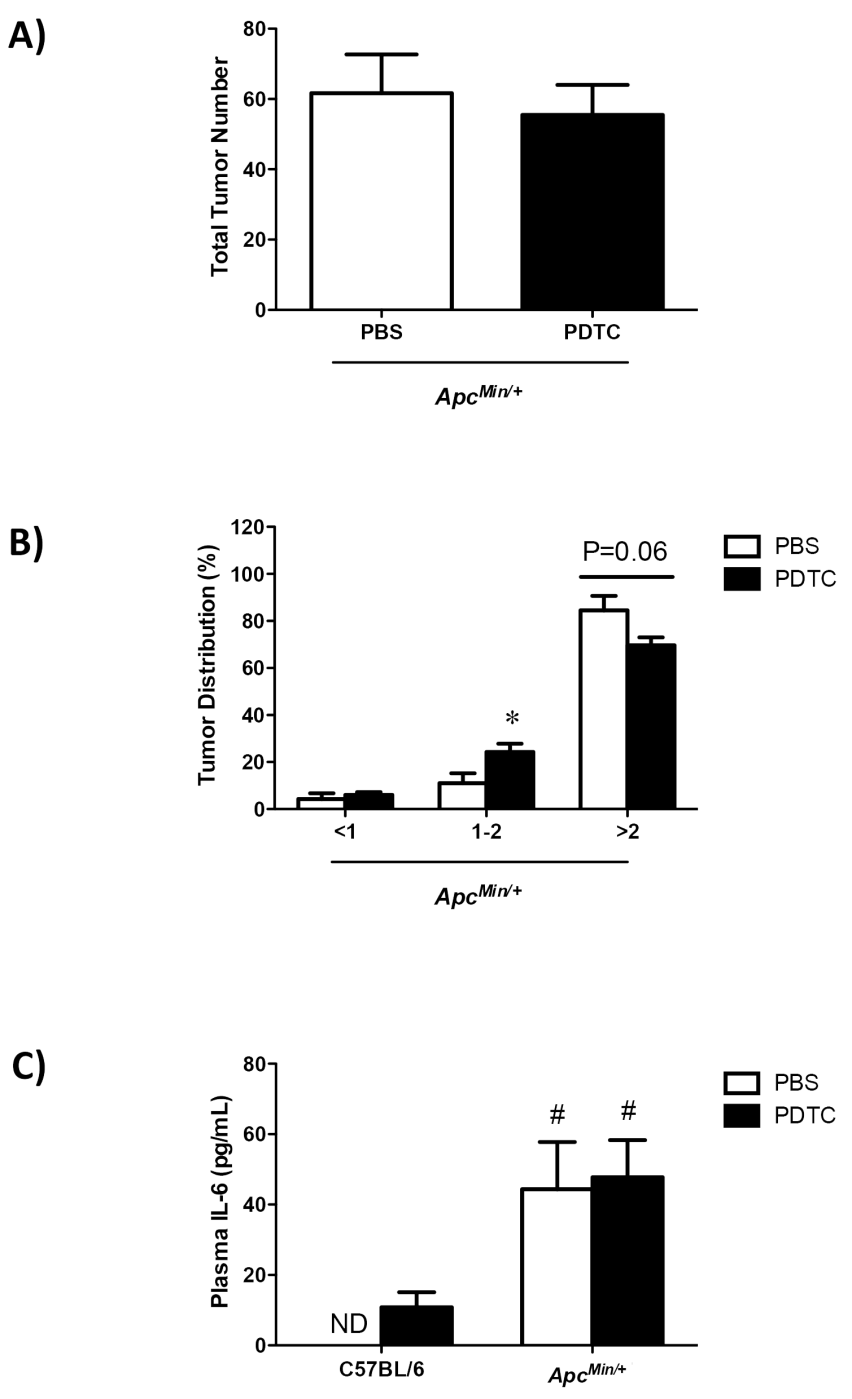

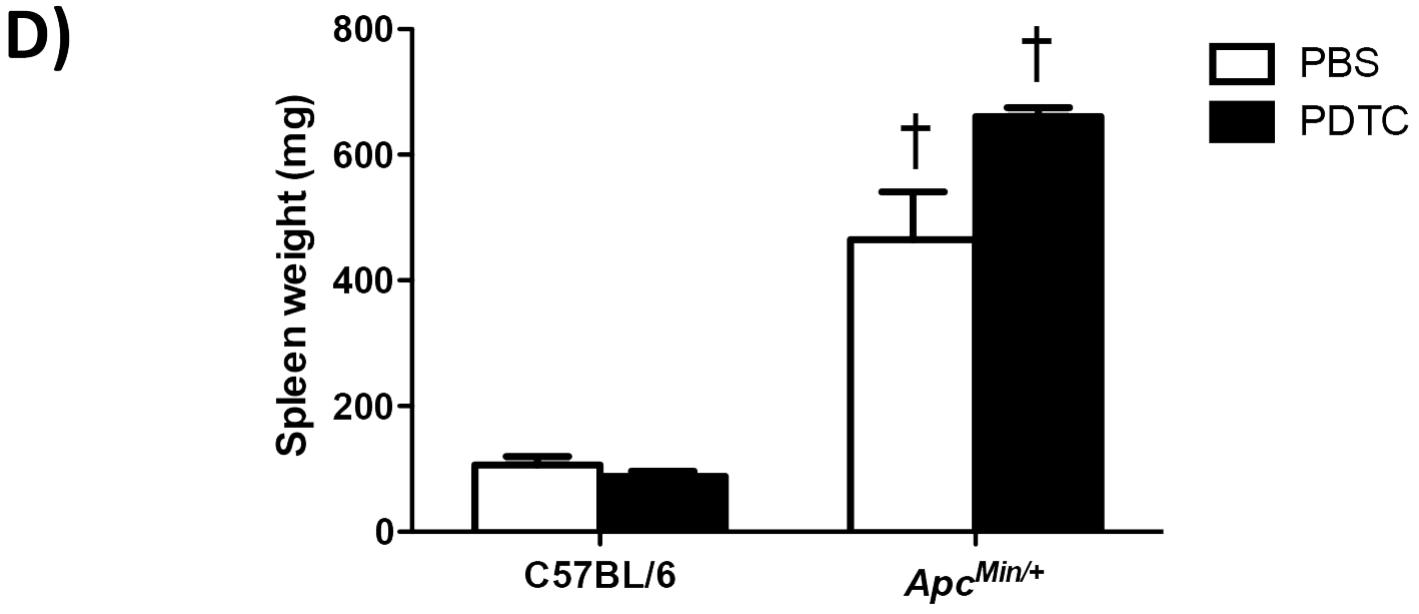
The effect of PDTC treatment on systemic inflammation in *Apc^Min/+^* mice **A.** Total tumor number in *Apc^Min/+^* mice. **B.** Tumor size distribution in *Apc^Min/+^* mice. **C.** Plasma IL-6 levels at the time of sacrifice in C57BL/6 and *Apc^Min/+^* mice. **D.** Spleen weight in C57BL/6 and *Apc^Min/+^* mice. Values are means ± standard error. Significance was set at P<0.05. † = signifies difference to all groups. * = signifies difference from PBS treatment within genotype. # = signifies main effect of *Apc^Min/+^* genotype. Abbreviations: pg, picogram. mL, milliliter. mg, milligram. PBS, phosphate buffered saline. PDTC, pyrrolidine dithiocarbamate. ND, not detected.

The effects of PDTC treatment on indices of systemic inflammation were examined in *Apc^Min/+^* mice. While systemic inflammation inhibition could affect tumor growth, PDTC treatment did not affect total tumor number in *Apc^Min/+^* mice (Figure 2A). Conversely, PDTC treatment increased the number of medium size (1-2mm) tumors and there was a strong trend (P=0.06) to decrease the number of large size (>2mm) tumors in *Apc^Min/+^* mice (Figure 2B). While alterations in tumor distribution could affect circulating cytokines, PDTC treatment did not alter the induction of plasma IL-6 in *Apc^Min/+^* mice (Figure 2C). Spleen weight was increased in *Apc^Min/+^* mice, and was further increased by PDTC treatment (Figure 2D). Collectively, these data demonstrate that while PDTC treatment attenuated body weight and muscle mass loss, systemic inflammation related to plasma IL-6 and total tumor number remained elevated regardless of PDTC treatment in *Apc^Min/+^* mice.

### Effect of PDTC treatment on muscle inflammatory signaling in *Apc^Min/+^* mice

**Figure 3:**
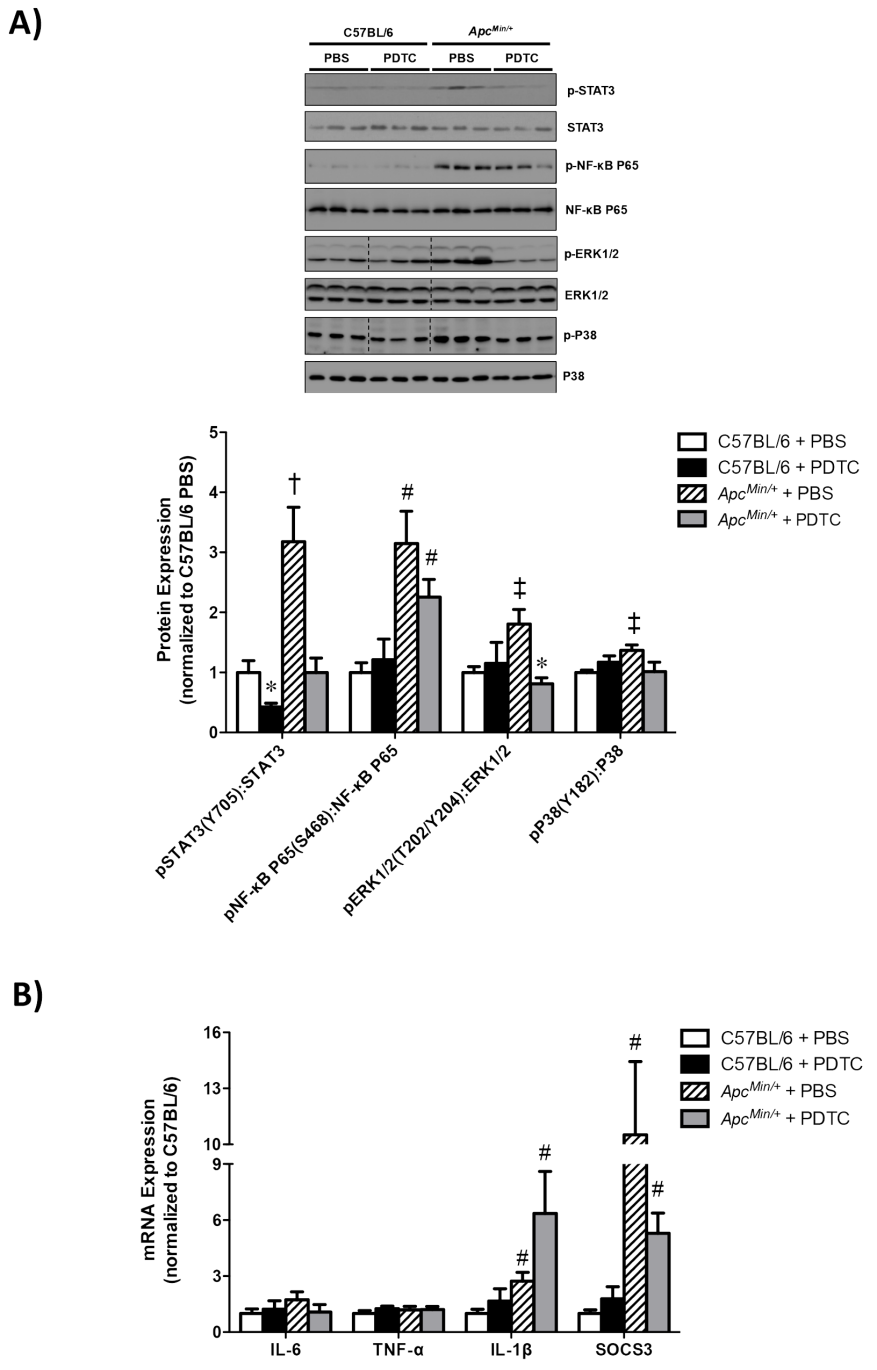
The effect of PDTC treatment on muscle inflammatory signaling and gene expression in *Apc^Min/+^* mice **A.** *Upper*. Representative Western blot of phosphorylated STAT3 (Y705), total STAT3, phosphorylated P65 (S468), total P65 phosphorylated ERK1/2 (T202/Y204), total ERK1/2, phosphorylated P38 (Y182), and total P38. *Lower*. The quantified ratio of phosphorylated to total STAT3, P65, ERK1/2, and P38 expression. Data are normalized to C57BL/6 + PBS mice. All samples were run on the same gel. Dotted lines demonstrate that images were cropped for representative purposes. **B.** Muscle inflammatory gene expression. Data are normalized to C57BL/6 + PBS mice. Values are means ± standard error. Significance was set at P<0.05. * = signifies difference from PBS treatment within genotype. † = signifies difference from all other groups. ‡ = signifies differences from C57BL/6** + PBS. # = signifies main effect of *Apc^Min/+^* genotype. Abbreviations: PBS, phosphate buffered saline. PDTC, pyrrolidine dithiocarbamate.

The effect of PDTC treatment on gastrocnemius muscle inflammatory signaling was examined in *Apc^Min/+^* mice. Muscle STAT3 (Y705) phosphorylation was induced in *Apc^Min/+^* mice, which was blocked by PDTC treatment (Figure 3A). In addition, PDTC treatment decreased STAT3 (Y705) phosphorylation in C57BL/6 mice. Conversely, PDTC treatment did not suppress the induction of NF-κB (S468) phosphorylation in *Apc^Min/+^* mice (Figure 3A). There was no effect of PDTC treatment on NF-κB (S468) phosphorylation in C57BL/6 mice. Muscle ERK1/2 (T202/Y204) phosphorylation was increased in *Apc^Min/+^* mice, which was blocked by PDTC treatment (Figure 3A). While muscle P38 (Y182) phosphorylation was increased in *Apc^Min/+^* mice, there was a trend (P=0.07) for PDTC treatment to decrease P38 (Y182) phosphorylation in *Apc^Min/+^* mice. These data demonstrate differential effects of PDTC treatment on muscle inflammatory signaling during the progression of cachexia. Moreover, alterations in muscle STAT3 signaling were independent to changes in NF-κB signaling in cachectic skeletal muscle.

We also examined the effect of PDTC treatment on gastrocnemius muscle inflammatory gene expression in *Apc^Min/+^* mice. As we have previously reported [31], muscle IL-6 gene expression was not altered in cachectic *Apc^Min/+^* mice, and there was no effect of PDTC treatment irrespective of genotype (Figure 3B). Additionally, there was no effect of cachexia or PDTC treatment on muscle TNF-α gene expression. Interestingly, we found that muscle IL-1β gene expression was increased in *Apc^Min/+^* mice, which was not altered with PDTC treatment (Figure 3B). Similar to our recent findings [32], muscle SOCS3 gene expression was highly induced in *Apc^Min/+^* mice, but there was no effect of PDTC treatment irrespective of genotype (Figure 3B). Since we have previously found that the activation of muscle inflammatory signaling and gene expression is dependent on the degree of cachexia, we examined the potential relationships between muscle gene expression and indices of cachexia. Interestingly, muscle IL-1β and SOCS3 gene expression were associated with several indices of cachexia, and several of these associations were altered by PDTC treatment (Table 2). Specifically, both muscle SOCS3 and IL-1β expression were associated with body weight loss, plasma IL-6, gastrocnemius mass, and spleen weight. PDTC treatment disrupted the relationship between muscle SOCS3 expression and body weight loss, but had no effect on the association with plasma IL-6, gastrocnemius mass, or spleen weight. Conversely, PDTC treatment disrupted the relationship between muscle IL-1β expression and body weight loss, gastrocnemius mass, and spleen weight, but had no effect on the association with plasma IL-6. Collectively, these data demonstrate that the induction of muscle inflammatory signaling and gene expression were closely related to several indices of cachexia severity, and PDTC treatment could disrupt these associations in *Apc^Min/+^* mice.

**Table 2:** The effect of PDTC treatment on the relationship between gastrocnemius mRNA expression and indices of cancer cachexia

Gene expression	Cachexia Indices	*Apc^Min/+^*			
		R^2^	p value	R^2^	p value
**SOCS3 mRNA**	Body weight, sacrifice	0.33	0.06	0.08	0.38
	% BW change, peak to post	0.55	<0.01	0.36	0.05
	% BW, pre to post	0.79	<0.001	0.08	0.39
	Plasma IL-6	0.85	<0.001	0.71	<0.001
	GAS mass	0.41	0.03	0.43	0.03
	Spleen weight	0.57	<0.01	0.55	<0.01
**IL-1**β **mRNA**	Body weight, sacrifice	0.63	<0.01	0.11	0.31
	% BW change, peak to post	0.59	<0.01	0.12	0.30
	% BW, pre to post	0.53	0.01	0.12	0.30
	Plasma IL-6	0.59	<0.01	0.61	<0.01
	GAS mass	0.69	<0.01	0.22	0.14
	Spleen weight	0.70	<0.01	0.31	0.07

Pearson correlations were performed to determine the relationship between factors. C57BL/6 + PBS were included in each *Apc^Min/+^* treatment group analysis. Abbreviations: PBS, phosphate buffered saline. PDTC, pyrrolidine dithiocarbamate. BW, body weight. GAS, gastrocnemius. SOCS3, suppressor of cytokine signaling 3. IL-6, interleukin-6. IL-1β, interleukin-1β.

### Effect of PDTC treatment on muscle protein turnover regulation in *Apc^Min/+^* mice

**Figure 4:**
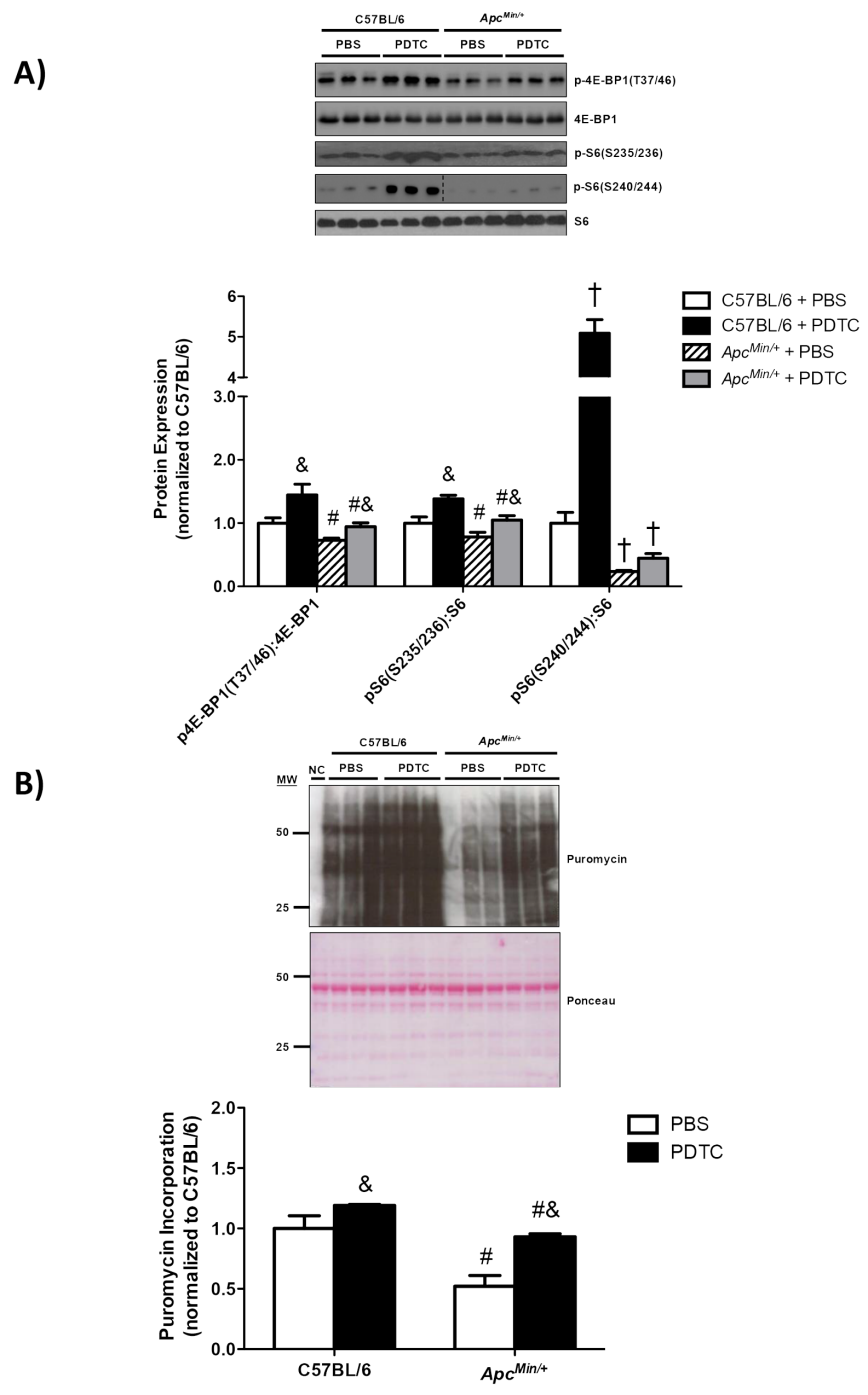

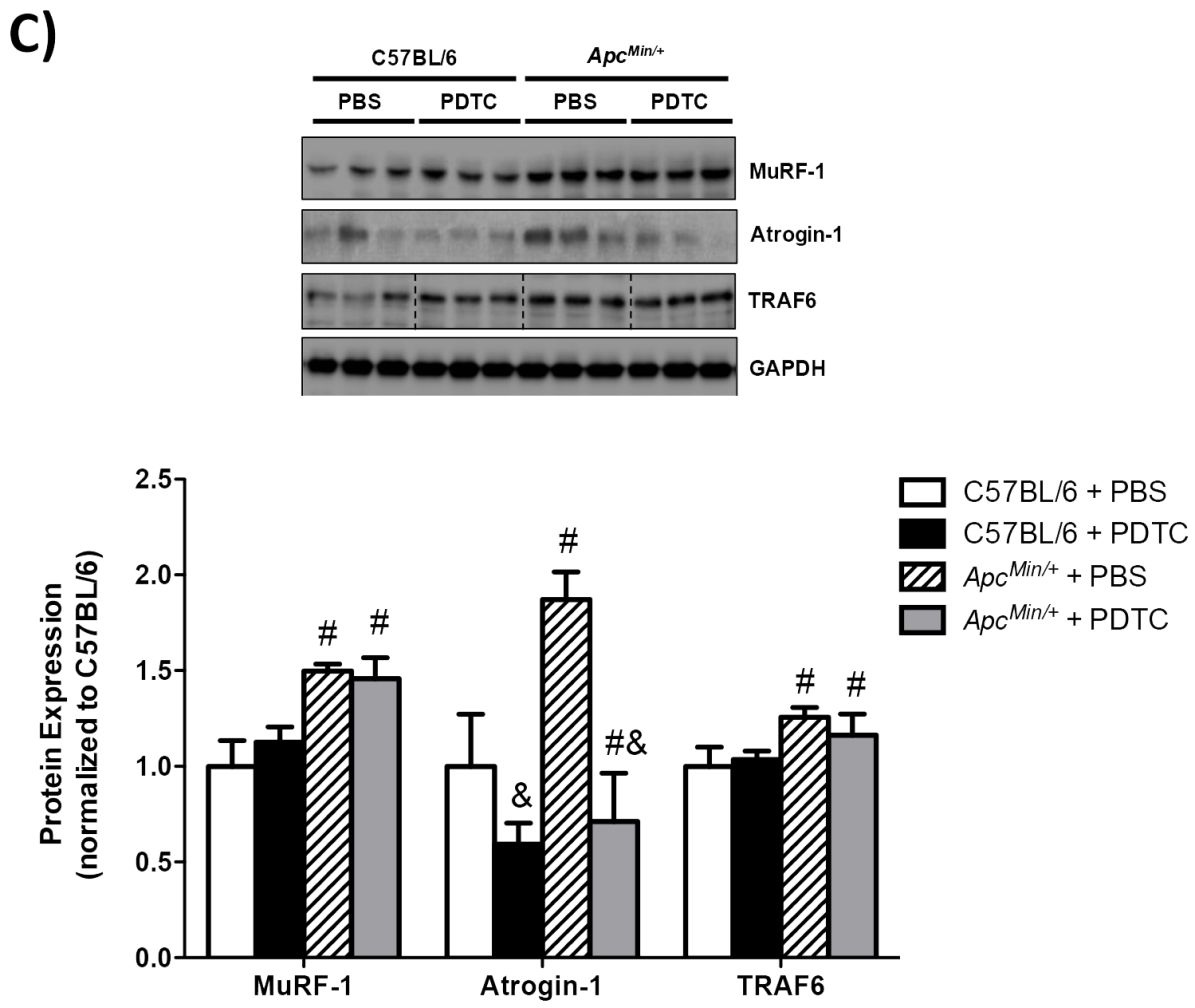
The effect of PDTC treatment on muscle protein turnover regulation in *Apc^Min/+^* mice **A.** *Upper*. Representative Western blot of phosphorylated 4E-BP1 (T37/46), total 4E-BP1, phosphorylated S6 (S235/236), phosphorylated S6 (S240/244), and total S6. *Lower*. The quantified ratio of phosphorylated to total 4E-BP1 and S6. Data are normalized to C57BL/6 + PBS mice. All samples were run on the same gel. Dotted lines demonstrate that images were cropped for representative purposes. **B.** *Upper*. Representative Western blot of total muscle puromycin incorporation. *Lower*. Quantification of total puromycin incorporation. Data are normalized to C57BL/6 + PBS mice. **C.** *Upper*. Representative Western blot of total MuRF-1, Atrogin-1, and TRAF6. *Lower*. The quantification of total MuRF-1, Atrogin-1, and TRAF6. Data are normalized to C57BL/6 + PBS mice. All samples were run on the same gel. Dotted lines demonstrate that images were cropped for representative purposes. Values are means ± standard error. Significance was set at P<0.05. † = signifies difference from all other groups. & = signifies main effect of PDTC treatment. # = signifies main effect of *Apc^Min/+^* genotype. Abbreviations: PBS, phosphate buffered saline. PDTC, pyrrolidine dithiocarbamate.

The effect of PDTC treatment on gastrocnemius muscle protein turnover was examined in *Apc^Min/+^* mice. To this end, we examined muscle mTORC1 signaling, protein synthesis, and E3 ligase protein expression. First we examined the phosphorylation of the direct mTORC1 target, 4E-BP1. Muscle 4E-BP1 (T37/46) phosphorylation was decreased in *Apc^Min/+^* mice, but PDTC treatment increased 4E-BP1 (T37/46) phosphorylation regardless of genotype (Figure 4A). We then examined two S6 phosphorylation sites which can be regulated by p70S6K, another direct mTORC1 target. Muscle S6 (S235/236) phosphorylation was decreased in *Apc^Min/+^* mice, which was increased by PDTC treatment regardless of genotype (Figure 4A). While muscle S6 (S240/244) phosphorylation was decreased by cachexia, there was only a trend (P=0.07) for PDTC treatment to increase muscle S6 (S240/244) phosphorylation in *Apc^Min/+^* mice. Interestingly, PDTC treatment increased muscle S6 (S240/244) phosphorylation in C57BL/6 mice. We next examined muscle protein synthesis by nonradioactive puromycin incorporation. Muscle protein synthesis was suppressed in *Apc^Min/+^* mice, and PDTC treatment increased muscle protein synthesis irrespective of genotype (Figure 4B). These data demonstrate that PDTC treatment improved muscle protein synthesis and mTORC1 signaling in *Apc^Min/+^* mice. We next examined the effects of PDTC treatment on the expression of several E3 ligases implicated in muscle proteolysis [33, 34]. MuRF-1 protein expression was increased in *Apc^Min/+^* mice, which was not affected by PDTC treatment irrespective of genotype (Figure 4C). In contrast, muscle Atrogin-1 protein expression was increased by cachexia, which was reduced by PDTC treatment regardless of genotype (Figure 4C). Lastly, TRAF6 protein expression was induced in *Apc^Min/+^* mice, but there was no effect of PDTC treatment (Figure 4C). Overall, these data demonstrate that reductions in STAT3 signaling, independent to NF-κB, were accompanied by improvements in protein turnover regulation during the progression of cancer cachexia in *Apc^Min/+^* mice.

### Effect of PDTC treatment on liver inflammatory signaling and protein synthesis in *Apc^Min/+^* mice

**Figure 5:**
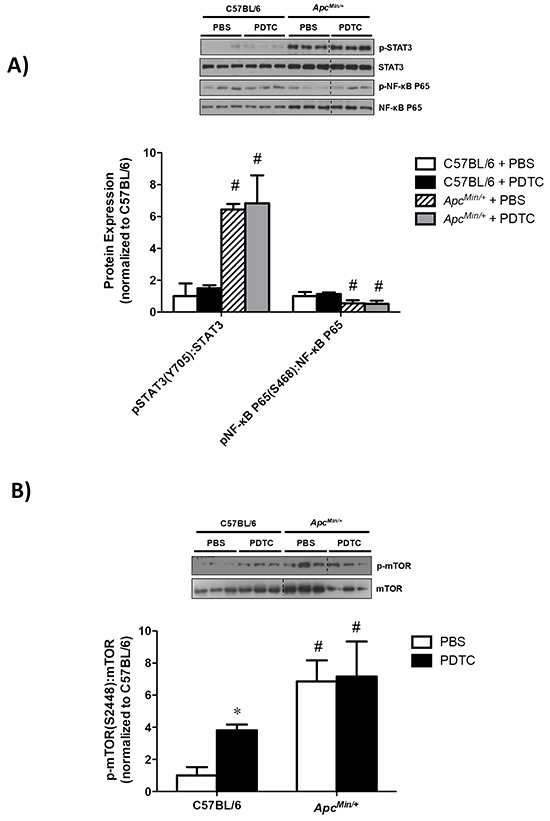

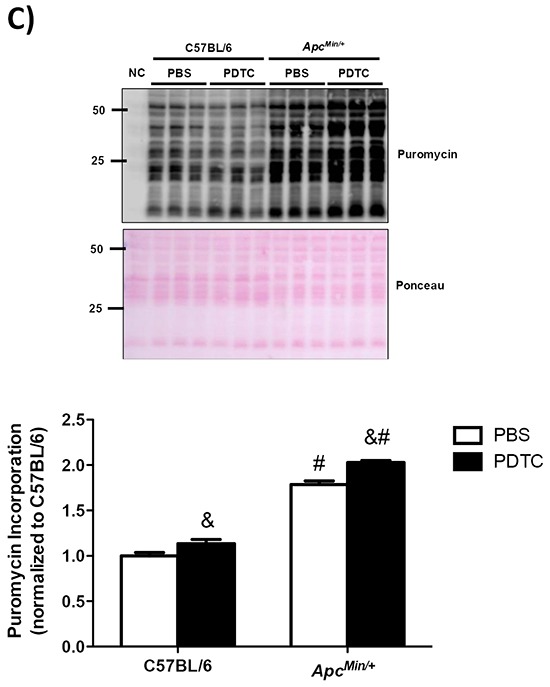
The effect of PDTC treatment on liver inflammatory signaling and protein synthesis in *Apc^Min/+^* mice **A.** *Upper*. Representative Western blot of phosphorylated STAT3 (S727), total STAT3, phosphorylated P65 (S468), and total P65. *Lower*. The quantified ratio of phosphorylated to total STAT3 and P65 expression. Data are normalized to C57BL/6 + PBS mice. All samples were run on the same gel. Dotted lines demonstrate that images were cropped for representative purposes. **B.** *Upper*. Representative Western blot of phosphorylated (S2448) and total mTOR expression. *Lower*. The quantified ratio of phosphorylated to total mTOR expression. Data are normalized to C57BL/6 + PBS mice. All samples were run on the same gel. Dotted lines demonstrate that images were cropped for representative purposes. **C.** *Left*. Representative Western blot of liver puromycin incorporation. *Lower*. The quantification of liver puromycin incorporation. Data are normalized to C57BL/6 + PBS mice. All samples were run on the same gel. Values are means ± stardard error. Significance was set at P<0.05. * = signifies difference from PBS within genotype. & = signifies main effect of PDTC treatment. # = signifies main effect of *Apc^Min/+^* genotype. Abbreviations: PBS, phosphate buffered saline. PDTC, pyrrolidine dithiocarbamate.

The effect of PDTC treatment on liver inflammatory signaling was examined in *Apc^Min/+^* mice. While liver STAT3 (S727) phosphorylation was increased in *Apc^Min/+^* mice, there was no effect of PDTC treatment irrespective of genotype (Figure 5A). In contrast, liver NF-κB (S468) phosphorylation was decreased in *Apc^Min/+^* mice, and there was no effect of PDTC treatment irrespective of genotype. Liver mTOR (S2448) phosphorylation was induced in *Apc^Min/+^* mice, and there was no effect of PDTC treatment (Figure 5B). Interestingly, mTOR (S2448) phosphorylation was increased by PDTC treatment in C57BL/6 mice. In line with mTOR (S2448) phosphorylation, liver protein synthesis rates were elevated in *Apc^Min/+^* mice, and PDTC treatment increased protein synthesis irrespective of genotype (Figure 5C). Collectively, these data demonstrate that PDTC treatment did not alter the differential activation of liver inflammatory signaling and disrupted mTOR phosphorylation during the progression of cachexia. In addition, these results highlight tissue specific signaling responses to PDTC treatment during cachexia progression.

### Effect of PDTC treatment on liver glycogen and lipid content in *Apc^Min/^+* mice

**Figure 6:**
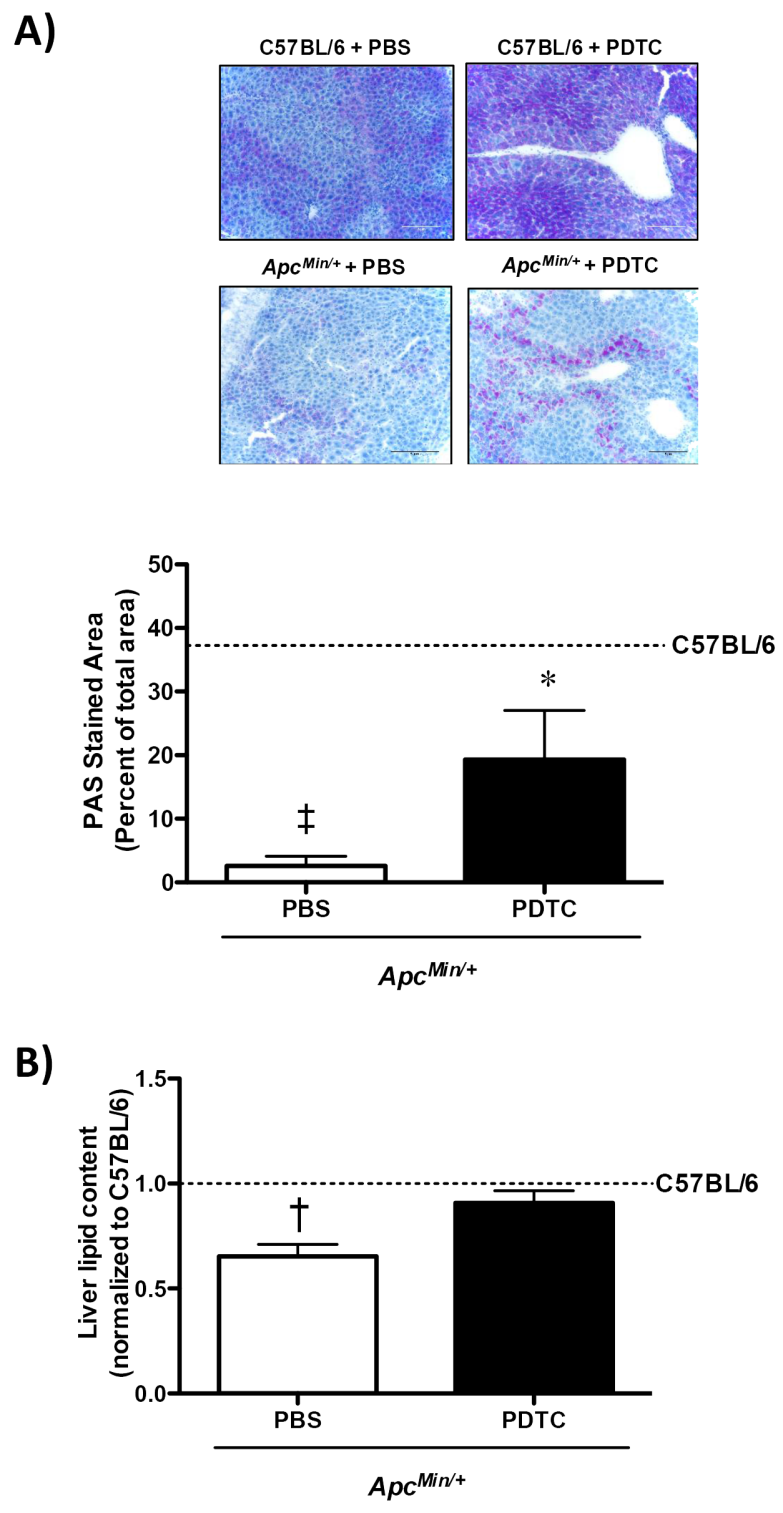
The effect of PDTC treatment on liver glycogen and lipid content in *Apc^Min/+^* mice **A.** *Upper*. Representative PAS stained liver. *Lower*. Quantification of liver glycogen content. **B.** Quantification of liver lipid content. Data are expressed as the percentage of C57BL/6 + PBS. Values are means ± standard error. Significance was set at P<0.05. ‡ = signifies differences from C57BL/6** + PBS. * = signifies difference from PBS within genotype. † = signifies difference from all other groups. Abbreviations: PAS, periodic acid-Schiff. PBS, phosphate buffered saline. PDTC, pyrrolidine dithiocarbamate.

The effect of PDTC treatment on liver glycogen and lipid content was examined in *Apc^Min/+^* mice. Liver glycogen content was reduced in *Apc^Min/+^* mice, which was attenuated by PDTC (Figure 6A). There was no effect of PDTC treatment on liver glycogen content in C57BL/6 mice. Moreover, PDTC treatment prevented the loss of liver lipid content in *Apc^Min/+^* mice (Figure 6B). There was no effect of PDTC treatment on liver lipid content in C57BL/6 mice. Collectively, these data demonstrate that while PDTC treatment did not alter liver inflammatory signaling, liver glycogen and lipid content were improved by PDTC treatment in *Apc^Min/+^* mice.

### Effect of PDTC treatment on liver metabolic gene expression in *Apc^Min/+^* mice

**Figure 7:**
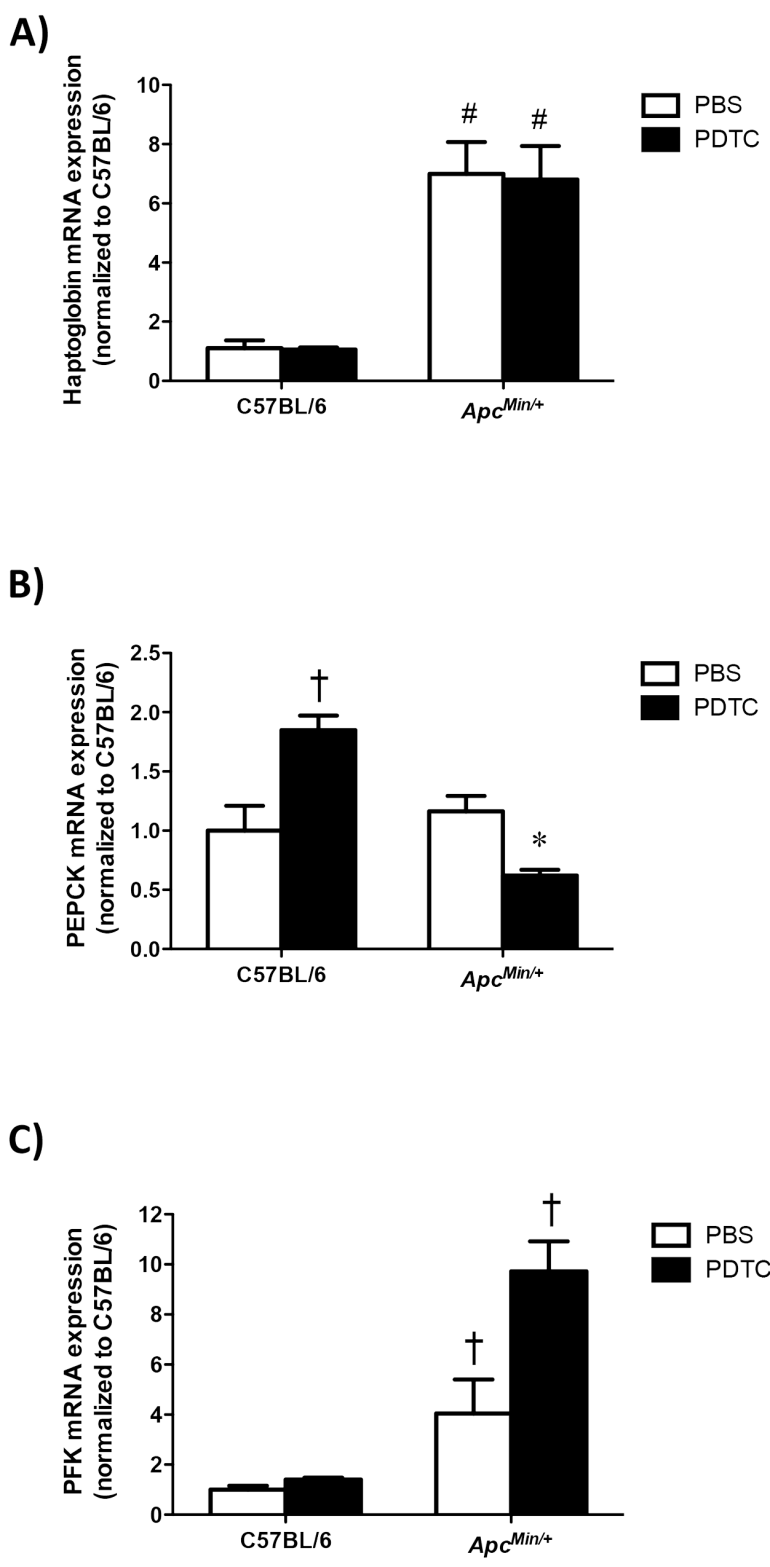
The effect of PDTC treatment on liver metabolic gene expression in *Apc^Min/+^* mice **A.** Quantification of liver haptoglobin gene expression. Data are normalized to C57BL/6 + PBS mice. **B.** Quantification of liver PEPCK gene expression. Data are normalized to C57BL/6 + PBS mice. **C.** Quantification of liver PFK gene expression. Data are normalized to C57BL/6 + PBS mice. Values are means ± standard error. Significance was set at P<0.05. * = signifies difference from PBS within genotype. † = signifies difference from all other groups. # = signifies main effect of *Apc^Min/+^* genotype. Abbreviations: PBS, phosphate buffered saline. PDTC, pyrrolidine dithiocarbamate. PEPCK, phosphoenolpyruvate carboxykinase. PFK, phosphofructokinase.

The effect of PDTC treatment on liver metabolic gene expression was examined in *Apc^Min/+^* mice. Liver haptoglobin gene expression increased in *Apc^Min/+^* mice, and there was no effect of PDTC treatment (Figure 7A). While PDTC treatment increased liver PEPCK gene expression in C57BL/6 mice, PEPCK gene expression was decreased by PDTC treatment in *Apc^Min/+^* mice (Figure 7B). Liver PFK gene expression was increased in *Apc^Min/+^* mice, which was further increased by PDTC treatment (Figure 7C). Overall, these data demonstrate that cachexia disrupts liver inflammatory signaling and metabolic gene expression, and metabolic gene expression is differentially affected by PDTC treatment in *Apc^Min/+^* mice.

## DISCUSSION

Systemic inflammation, muscle wasting, and liver metabolic dysfunction are hallmarks of cancer cachexia. Many studies targeting systemic inflammation inhibition have examined cachexia prevention in preclinical models, and have observed attenuation of muscle mass loss [24, 27, 35]. However, far fewer studies have been designed to treat the cachectic condition, with treatments initiated after the development of cachexia, which has clinical significance since many cancer patients are cachectic at the time of diagnosis [1, 26]. Additionally, many studies have focused on attenuated muscle mass loss through catabolic signaling, while forgoing the complexity of examining multiple target tissues and anabolic processes that also contribute to the complicated multifactorial nature of cachexia. While several preclinical models of cancer cachexia are available, the *Apc^Min/+^* mouse model of cancer cachexia exhibits chronically elevated plasma IL-6 levels, muscle wasting, and liver metabolic dysfunction [27, 28, 36]. Additionally, due to the long duration of cachexia development in the *Apc^Min/+^* mouse, studies can be designed to examine the treatment effects after the development of cachexia. The current study examined the effects of short-term PDTC treatment on disrupted skeletal muscle and liver signaling in cachectic *Apc^Min/+^* mice. PDTC treatment reduced muscle inflammatory signaling and improved protein turnover related to increased muscle protein synthesis and decreased Atrogin-1 protein expression. PDTC treatment also improved liver glycogen and lipid content independent to alterations in STAT3 signaling. Interestingly, these tissue specific changes occurred independent to total tumor number and circulating IL-6 levels, which were not affected by PDTC treatment. Collectively, these results demonstrate that short-term PDTC treatment can reverse several indices of cachexia progression independent to the suppression of chronic inflammation in tumor-bearing mice. Based on the short-term effects of PDTC treatment on several indices of cachexia progression, further studies are warranted to determine if the identified signaling pathways can improve long-term survival and maintain muscle and liver function in tumor bearing mice.

The maintenance of skeletal muscle mass is critically important to reduce morbidity and mortality in the cancer patient. In the current study, PDTC treatment improved hindlimb muscle mass during the progression of cachexia. Skeletal muscle mass is regulated by the rates of protein synthesis and degradation, which is disrupted throughout the progression of cancer cachexia. Cancer cachexia-induced muscle wasting is accompanied by the suppression of protein synthesis and activation of protein degradation [19, 20, 27]. While few therapies have been shown to increase muscle protein synthesis during the progression of cachexia, we recently found that a single PDTC dose increased muscle protein synthesis signaling in cachectic mice [20]. We have extended these findings by demonstrating PDTC treatment increased muscle protein synthesis in mice initiating cachexia. These findings are in contrast to systemic IL-6 inhibition, as an IL-6 receptor antibody was unable to rescue the suppression of muscle protein synthesis during the progression of cancer cachexia [27]. However, PDTC treatment has been shown to increase global protein translational capacity in non-muscle cells through a rapamycin independent mechanism [15]. Whether this mechanism occurs in skeletal muscle requires further investigation. We have previously found the induction of MuRF-1 and Atrogin-1 gene expression during cachexia progression, and systemic IL-6 inhibition can suppress muscle protein degradation in *Apc^Min/+^* mice [27]. Moreover, we have previously shown PDTC treatment can block LLC-induction of Atrogin-1 protein expression in C2C12 myotubes [21]. In the current study, we found that several E3 ligases were induced by cancer cachexia, but PDTC treatment only reduced muscle Atrogin-1 protein expression in *Apc^Min/+^* mice. These findings may be related to NF-κB regulation of proteolytic gene expression, which was not completely blocked by PDTC treatment in the current study. Nonetheless, improvements in muscle protein turnover occurred despite the presence of a cachectic systemic environment. Further work is needed to mechanistically determine the role of PDTC treatment on muscle protein turnover regulation during the progression of cachexia.

Muscle STAT3 has documented roles in both muscle atrophy and hypertrophy processes [29, 35, 37–40]. While muscle STAT3 contributes to muscle wasting in several models of cancer cachexia [27, 29, 35, 41], this pathway is also activated in rodent models of muscle growth [39, 40, 42, 43]. Interestingly, recent evidence also suggests STAT3 signaling can also regulate basal muscle mass in the absence of anabolic or catabolic stimuli. For example, STAT3 knockdown by *in vivo* electroporation for 2 weeks was sufficient to induce tibialis anterior myofiber growth in healthy mice [35]. In the current study, we found that PDTC treatment reduced STAT3 signaling, which was accompanied by muscle growth and mTORC1 activation in both healthy and cachectic mice. Regardless of cachexia, PDTC treatment promoted an anabolic shift in muscle protein turnover through an induction in muscle mTOR signaling and protein synthesis, and reductions in Atrogin-1 protein expression. Intriguingly, reduced muscle STAT3 activity was independent to alterations in NF-κB signaling. While these findings demonstrate a unique role for STAT3 in the regulation of basal muscle protein turnover, the precise mechanism by which STAT3 regulates these processes remains to be determined. Moreover, since STAT3 signaling pathways can be induced by both catabolic and anabolic stimuli, further research is needed to mechanistically determine STAT3 regulation of muscle mass in response to diverse stimuli.

Systemic inflammation associated with cancer can disrupt liver metabolic function, which can contribute to the wasting process during the progression of cancer cachexia [28, 29, 44, 45]. Similar to muscle, liver STAT3 is elevated in the cachectic mice. While enhanced muscle STAT3 activity corresponds to the suppression of protein synthesis, liver protein synthesis is elevated during the progression of cachexia [28] and can promote hepatomegaly in cachectic mice [28, 29]. It has been proposed that the induction of liver protein synthesis may support acute phase protein (APP) production, and contributes to the hypermetabolic state and wasting process [6]. Interestingly, these alterations in protein synthesis and liver hypertrophy can occur despite the depletion of lipid and glycogen stores [28]. In the current study, PDTC treatment was unable to suppress chronically active liver mTOR, protein synthesis, and hypertrophy. Unexpectedly, PDTC treatment led to liver hypertrophy independent of the genotype. PDTC treatment may have increased liver growth through the induction of mTOR signaling in healthy mice, but this requires further investigation. Related to cachexia, we have previously found reduced liver glycogen content during cachexia progression [28]. We extend these findings and further demonstrate PDTC treatment improved glycogen and lipid content. Previous studies have found improved hepatic glycogen content and suppressed liver gluconeogenic enzyme expression by PDTC treatment in diabetic rats [46]. We extend these findings by showing that PDTC suppressed the liver gluconeogenic enzyme PEPCK in cachectic *Apc^Min^^/+^* mice. Interestingly, suppressed PEPCK mRNA expression was associated with increased expression of the glycolytic enzyme PFK, which suggests PDTC altered glucose metabolism in the cachectic liver. Whether these changes in metabolic gene expression were associated with improved cachectic liver function remains to be determined. Additionally, further studies are required to mechanistically determine the role of inflammatory signaling inhibition on liver metabolism during the progression of cancer cachexia.

## CONCLUSIONS

In conclusion, these results demonstrate that short-term PDTC treatment to the cachectic mouse is sufficient to rescue cancer-induced disruptions to muscle and liver signaling, and these changes are independent of changes in tumor burden and circulating IL-6. This study also highlights the contrasting effects of a single therapy on two different organs disrupted during cachexia progression. Improvements in muscle mass were accompanied by suppressed inflammatory signaling, attenuated protein degradation, and activated protein synthesis. Furthermore, PDTC treatment increased liver glycogen and lipid content independent to alterations in inflammatory signaling. These tissue specific responses were independent of total tumor number and circulating IL-6. While these data suggest PDTC may have therapeutic potential to combat cachexia associated with cancer, further research is required to determine its efficacy due to hypertrophy of visceral organs such as the heart and liver; which may elicit a false positive for lean mass measurements by DEXA. Moreover, the interactions between inflammatory signaling and therapeutic countermeasures such as nutrition and physical activity on cachexia progression should be examined. Further work is required to mechanistically determine if this type of treatment can fully rescue suppressed metabolic functions of cachectic muscle and liver, and lead to improved survival.

## MATERIALS AND METHODS

### Animals

*Apc^Min/+^* mice on a C57BL/6 background were originally purchased from Jackson Laboratory (Bar Harbor, ME, USA) and bred at the University of South Carolina's Center for Colon Cancer Research Mouse Core in the primary investigators breeding colony. Mice were housed in standard cages and kept on a 12:12hr light-dark cycle with the light period starting 0700 hrs. Mice were provided standard rodent chow (cat #8604, Harlan, Teklad Rodent Diet, Madison, WI) and water *ad libitum*. Two cohorts of male C57BL/6 and *Apc^Min/+^* mice were weighed weekly and monitored for qualitative signs of cachexia progression (morbidity, voluntary activity, grooming behavior). The first cohort of mice (C57BL/6 + PBS: N=5, C57BL/6 + PDTC: N=5, *Apc^Min/+^* + PBS: N=6, *Apc^Min/+^* + PDTC: N=6) was used for all analysis throughout the manuscript (excluding food intake), while the second cohort of mice (C57BL/6 + PBS: N=5, C57BL/6 + PDTC: N=6, *Apc^Min/+^* + PBS: N=5, *Apc^Min/+^* + PDTC: N=7) was included post-hoc to determine the effects of PDTC treatment on food intake. At 16-18 weeks of age C57BL/6 and *Apc^Min/+^* mice were randomized to either phosphate-buffered saline (PBS) or pyrrolidine dithiocarbamate (PDTC) treatment based on the percentage body weight loss from peak-measurement. Both cohorts of *Apc^Min/+^* mice had initiated cachexia prior to the treatment period. All animal procedures were approved by the University of South Carolina's Institutional Animal Care and Use Committee.

### Pyrrolidine dithiocarbamate (PDTC) administration

PDTC, a STAT3 and NF-κB inhibitor (Sigma-Aldrich, cat #P8765, St. Louis, MO), was reconstituted in sterile phosphate buffer saline (PBS) to a stock concentration of 100 mg/ml and stored at 4°C. PDTC (10 mg/kg body weight) or PBS was administered daily via an intraperitoneal injection for 2 weeks (Figure 1A). All treatment groups were sacrificed 18 hrs after the last treatment.

### Cage activity monitoring

Voluntary cage activity was monitored as previously described [20, 47]. Mice were single-housed in activity monitor cages (Opto-M3 Activity Meter, Columbus Instruments) and spontaneous activity was measured for 10 h during the dark cycle. The number of beams crossed in the X-Y plane were recorded and averaged for two consecutive nights prior to sacrifice.

### Tissue collection

Mice were sacrificed during the light cycle following a 5 hour fast. Thirty minutes prior to sacrifice all mice received an intraperitoneal injection of puromycin (0.040 μmol/g body weight, cat #540411, Calbiochem, San Diego, CA) in warm sterile PBS [19, 48]. Fifteen minutes prior to tissue collection mice were anesthetized by a subcutaneous injection of the ketamine/xylazine/acepromazine cocktail (1.4 ml/kg body weight). Once anesthetized a dual-energy X-ray absorptiometry (DEXA) scan was performed to determine total fat and fat free mass. Blood was then collected via retro-orbital sinus. Exactly 30 minutes after the puromycin injection hind limb muscles and organs were excised, cleared of connective tissue, weighed and snap frozen in liquid nitrogen. Intestinal segments were excised, cleaned with PBS, cut into equal segments, and stored in 10% neutral formalin until tumor count analysis. Total polyp counts and distributions were performed by an investigator blinded to the treatment groups as previously described [49–51]. Blood was centrifuged at 10,000 x g for 10 minutes at 4°C. Plasma and tissue samples were stored at −80°C until analysis.

### Plasma IL-6 levels

Plasma IL-6 concentrations were quantified using a standard mouse IL-6 ELISA kit (BD BioSciences, cat #550950, San Jose, CA). Briefly, 25-50ul of plasma was diluted to a total volume of 100ul and IL-6 concentrations (pg/mL) were determined in duplicate according to manufacturer's instructions. The lower limit of detection for this assay was 3.8 pg/mL.

### RNA isolation, cDNA synthesis and real-time PCR

RNA isolation, cDNA synthesis and real-time PCR were performed as described previously [34]. Briefly, RNA was isolated by homogenizing muscle and liver tissue in Trizol (Invitrogen, cat#15596, Carlsbad, CA) followed by a chloroform/isopropyl alcohol extraction. cDNA synthesis and RT-PCR was performed using reagents purchased from Applied Biosystems (ABI, Foster City, CA). Primers for suppressor of cytokine signaling 3 (SOCS3) [29], haptoglobin [29], phosphofructokinase (PFK) [52], phosphoenolpyruvate carboxykinase (PEPCK) [52], IL-6 [24], tumor necrosis factor-α (TNF-α), and interleukin-1β (IL-1β) were purchased from IDT (Coralville, IA). Primer sequences were as follows: TNF-α forward, 5′-CCCAGACCCTCACACTCAGAT-3′, and TNF-α reverse, 5′-TTGTCCCTTGAAGAGAACCTG-3′ and IL-1β forward, 5′-ATCGCAACTGTTCCTGAACTCAACT-3′ and IL-1β reverse, 5′-CAGGACAGGTATAGATTCTTTCCTTT-3′. Data was analyzed using the comparative cycle threshold [Ct] method calculated by the ABI software.

### Western blot

Western blots were performed as described previously [32, 53]. Briefly, approximately 30 mg of frozen liver or muscle tissue was cut, weighed, and homogenized in Mueller buffer using a glass on glass homogenizer. Homogenates were spun at 13,000 rpm for 10 minutes at 4°C and the resulting supernatant was quantified for protein concentration using the Bradford assay (Bio-Rad, cat #500-0006, Hercules, CA). Crude homogenates were fractionated on 6-15% SDS-polyacrylamide gels and transferred to a polyvinylidene fluoride membrane overnight. Membranes were stained with Ponceau red to verify equal loading and transfer of each gel. Membranes were blocked for 1 hour at room temperature (RT) in 5% non-fat milk in Tris-buffered saline with 0.1% Tween-20 (TBST) or phosphate buffered saline (PBST) and then incubated with primary antibodies against STAT3 (Y705) (cat #4113), STAT3 (S727) (cat #9134), STAT3 (cat #4904), P65 (S468) (cat #3039), P65 (cat #4764), ERK1/2 (T202/Y204) (cat #9101), ERK1/2 (cat #4348), P38 (Y182) (cat #sc-7975, Santa Cruz Biotechnology, Dallas, TX), P38 (cat #sc-7972, Santa Cruz Biotechnology, Dallas, TX), TRAF6 (cat#: sc-7221, Santa Cruz Biotechnology, Dallas, TX), MuRF-1 (cat#: MP3401, ECM Biosciences, Versailles, KY), Atrogin-1 (cat #AP2041, ECM Biosciences, Versailles, KY), S6 (S235/236) (cat #2211), S6 (240/244) (cat #2215), S6 (cat #3039), mTOR (S2448) (cat #2971), mTOR (cat #2972), Puromycin (cat #MABE343, Millipore, Billercia, MA), and GAPDH (cat #2118) diluted in 5% milk-TBST. Anti-mouse (cat #7076) and anti-rabbit (cat #7074) IgG horseradish-peroxidase conjugated secondary antibody was incubated with membranes at 1:2000 dilutions for 1 hour in 5% milk-TBST. Primary and secondary antibodies were purchased from Cell Signaling Technology (Danvers, MA) unless otherwise stated. Enhanced chemiluminescence (Quantum ECL, BioExpress, Kaysville, UT) was used to visualize the antibody-antigen interaction. Blots were acquired using film (CL-Xposure, Thermo Scientific, Waltham, MA) or digital imaging (G:Box, Syngene, Frederick, MD), and quantified by densitometry using imaging software (ImageJ, NIH, Bethesda, MD).

### Periodic acid Schiff's staining

A small piece of liver tissue was mounted in optimal cutting tissue (OCT) and sectioned at a thickness of 12μm on a cryostat at -20°C. The sections were fixed in Carnoy's fixative for 10 minutes followed by a 30-minute incubation in Periodic Acid. Sections were then washed with dH_2_O and incubated in Schiff's reagent for 30 minutes. The sections were counter stained with hematoxylin, dehydrated through graded alcohol, and mounted using Permount. Sections were imaged the next day using an Olympus microscope with a DP70 CCD camera (Olympus, Centre Valley, PA). The percent PAS stained area was quantified using Image J software [54].

### Lipid extraction

Approximately 100 mg of frozen liver tissue was weighed and added to a tube containing 4ml of Chloroform:methanol mixture (2:1). The tissue was homogenized and the resulting mixture was gently mixed for 20 minutes. The homogenate was centrifuged to separate the organic and aqueous phases, and the bottom layer was carefully removed into a new pre-weighed 5 ml glass tube and evaporated using a nitrogen vacuum system. The dried tube was weighed and lipid content was calculated as the difference between the pre- and post-evaporation tube weights [55].

### Statistical analysis

Statistical analysis was performed using GraphPad (V5, La Jolla, CA, USA) and Sigma Stat software (V3.5, San Jose, CA, USA). A two-way ANOVA was used to determine differences between genotype and PDTC treatment. Post-hoc analyses were performed with Student Newman-Keul methods. Differences in total polyp number and distribution were determined by a student's t-test. Pearson correlations were performed to examine associations between muscle gene expression and indices of cancer cachexia. Values were expressed as mean ± standard error. Significance was set at p<0.05.

## SUPPLEMENTARY FIGURE


