# Supplementary material for: Short-term pyrrolidine dithiocarbamate administration attenuates cachexia-induced alterations to muscle and liver in ApcMin/+ mice
Source: Oncotarget. 2016 Jul 19;7(37):59482–502. doi: 10.18632/oncotarget.10699 (PMC5312326; doi:10.18632/oncotarget.10699)
Supplement: Supplementary file 1 [file oncotarget-07-59482-s001.pdf]

Short-term pyrrolidine dithiocarbamate administration attenuates cachexia-induced alterations to muscle and liver in *Apc*$^{Min/+}$ mice

SUPPLEMENTARY FIGURE

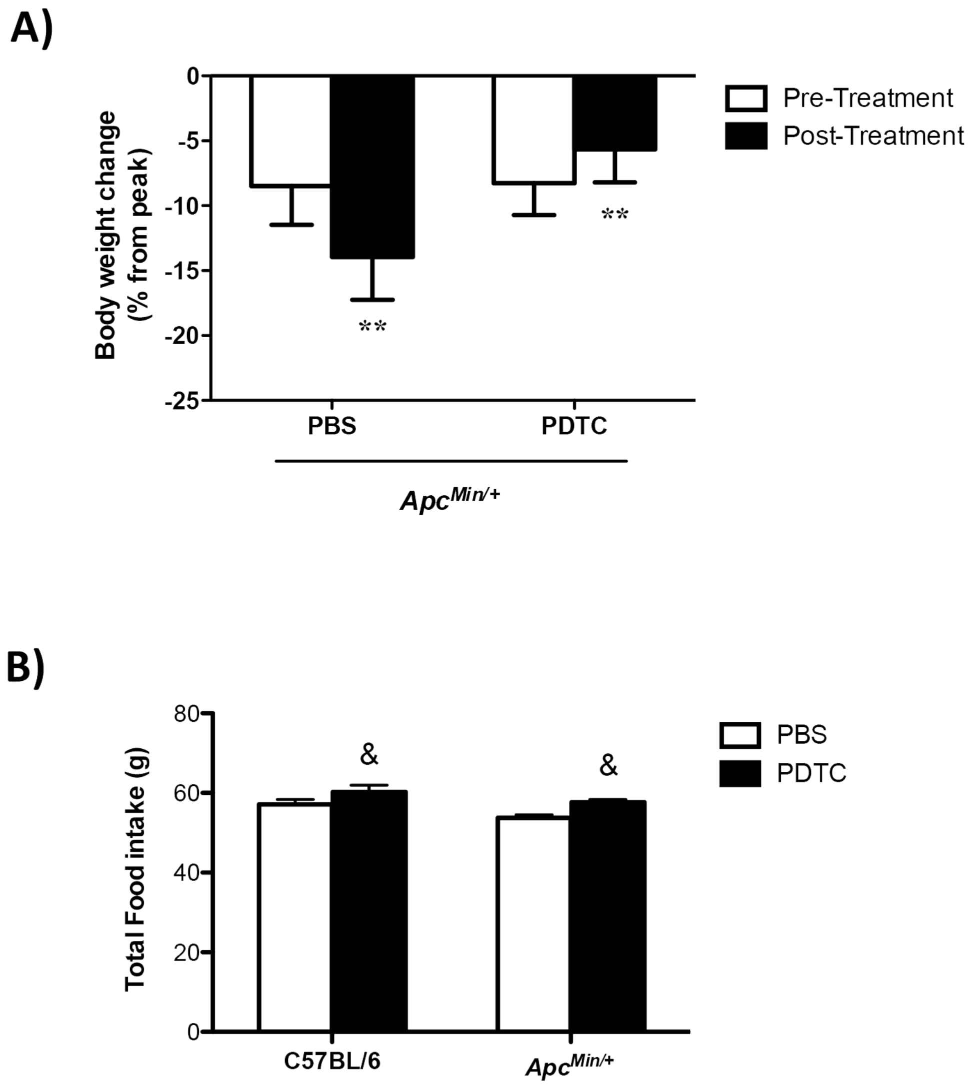

Supplementary Figure S1: The effect of PDTC treatment on food intake in *Apc*$^{Min/+}$ mice. A. The percentage body weight loss pre- and post-treatment in *Apc*$^{Min/+}$ mice. At 18 weeks of age C57BL/6 and *Apc*$^{Min/+}$ mice were randomized to receive PBS or PDTC (10 mg/kg body weight/day) via intraperitoneal injection for 2 weeks. Mice were sacrificed at 20 weeks of age following a 5 hr fast. **B.** Total food intake over the 2-week treatment period in C57BL/6 and *Apc*$^{Min/+}$ mice. C57BL/6 + PBS: N=5, C57BL/6 + PDTC: N=6, *Apc*$^{Min/+}$ + PBS: N=5, *Apc*$^{Min/+}$ + PDTC: N=7. Values are means ± standard error. Significance was set at $P<0.05$. & = signifies main effect of PDTC treatment. ** = signifies different to pre-treatment. Abbreviations: PBS, phosphate buffered saline. PDTC, pyrrolidine dithiocarbamate. g, grams.
